# Fabrication and Self-Assembly Behavior of BPEF and BBPEF Composite Langmuir–Blodgett Films with Photovoltaic Conversion Properties

**DOI:** 10.3390/nano14181514

**Published:** 2024-09-18

**Authors:** Feifei Wang, Lei Ge, Lin Li, Tianyue Zhao, Tifeng Jiao

**Affiliations:** 1 Hebei Key Laboratory of Pollution Prevention Biotechnology, School of Environmental Science and Engineering, Hebei University of Science and Technology, Shijiazhuang 050018, China; 2 State Key Laboratory of Metastable Materials Science and Technology, Yanshan University, Qinhuangdao 066004, China

**Keywords:** Langmuir–Blodgett, BPEF, BBPEF, dye, photoresponsive

## Abstract

The LB films prepared through the Langmuir–Blodgett (LB) technique are of significant importance for the fabrication of functional films such as optoelectronic materials and sensors. In this study, 9,9-bis (4-(2-hydroxy-ethoxy) phenyl) fluorene (BPEF) and 9,9-bis [3-phenyl-4-(β-hydroxy-ethoxy) phenyl] fluorene (BBPEF) were combined with saffron T (ST), methylene blue (MB) and Rhodamine B (RhB) dyes by LB technique to prepare ordered composite films. The nanostructures and morphologies of the composite films were analyzed by transmission electron microscopy (TEM) and atomic force microscopy (AFM). It was found that the films exhibited distinct aggregation morphologies. The UV-VIS absorption spectra showed that the concentration of dye molecules had a significant effect on the spectral characteristics. The contact Angle test shows that the prepared composite films are hydrophobic. The photovoltaic conversion performance of LB composite films was studied by transient photocurrent response experiments. It was found that BPEF/dye and BBPEF/dye composite films exhibited significant responses in photocurrent. In particular, BPEF/RhB and BBPEF/RhB composite films demonstrated excellent photoresponsive performance. This study used LB technology in combination with BPEF and BBPEF to demonstrate enhanced photocurrent and stable performance of LB film, which provided ideas for expanding the application range of materials.

## 1. Introduction

The Langmuir–Blodgett (LB) technique, derived from research by Langmuir and Katherine Blodgett according to academic standards [1,2], is a film-making technology that uses a controlled assembly of organic molecules and transfers a monolayer of organic molecules from the air/water interface to the solid substrate [3,4]. LB technology can control film thickness, prepare single or multilayer films [5], accumulate different molecular layers using polymer materials [6], and control molecular orientation [7,8]. The advantages mentioned above have made LB technology essential for producing nanoscale composite films. Furthermore, the thin films prepared by LB technology have a high degree of geometric structure order and excellent photoelectrochemical properties [9,10] and are widely used in optoelectric functional devices, biological and gas sensing film elements, modified electrodes, and other fields [11,12].

Dye molecules possess excellent optical properties and are often used as functional materials [13,14]. Saffron T (ST) is a commonly used water-soluble organic dye in both industry and scientific research [15,16]. Rhodamine B (RhB) demonstrates good solubility and stability under various solvent and environmental conditions and is widely used in a variety of applications [17]. Methylene blue (MB) is a typical cationic dye with good solubility and stability [18]. Due to the diversity of functional groups and low cost, dyes have become the key raw materials for the preparation of nanocomposites [19].

In addition, 9,9-bis (4-(2-hydroxy-ethoxy) phenyl) fluorene (BPEF) and its epoxidation derivative 9,9-bis [3-phenyl-4-(β-hydroxy-ethoxy) phenyl] fluorene (BBPEF) exhibit unique physicochemical properties and have a certain potential for manufacturing high-performance LB composite films. BPEF has a Cardo skeleton structure, which gives it good thermal stability and a thermal decomposition temperature of over 300 °C. This makes BPEF suitable for applications where thermal sensitivity is critical and improves the mechanical strength of the material [20]. BPEF exhibits excellent photoluminescent properties. Due to the strong conjugation effect of the fluorene group in its molecular structure, BPEF often shows high quantum efficiency, blue light emission, and high luminescence stability. Due to its fluorene structure, BPEF has high electrochemical stability and good electron transport capabilities. This gives it potential applications in organic electronic devices. BPEF has good solubility and dissolves in various organic solvents, which facilitates the preparation and processing of thin films. This makes BPEF well-suited for the manufacturing of organic electronic devices. BPEF has attracted wide attention in the field of organic optoelectronic materials due to its excellent optical and electronic properties [21]. The derivative BBPEF introduced a phenyl group, increasing the crosslinking density and giving the refractive index nD (25 °C) a value of up to 1.66. This structural change not only enhances the optical properties of the material but also improves its electron mobility in high-technology applications such as airfield-effect transistors and organic light-emitting diodes. This opens up new pathways for the application of LB composite films in specific functional areas.

Here, we employed the LB film technique to combine BPEF and BBPEF molecules with three dyes, ST, RhB, and MB, successfully preparing well-ordered BPEF/dye and BBPEF/dye interfacial self-assembled composite films. This study demonstrated that BPEF/RhB and BBPEF/RhB composite films exhibit excellent photoresponsive performance, providing research insights and methods for developing multifunctional thin-film electronic devices.

## 2. Experimental Section

### 2.1. Materials

Rhodamine B (RhB), anhydrous ethanol (EtOH), methylene blue (MB), and saffron T (ST) were purchased from Shanghai Aladdin Co., Ltd. (Shanghai, China). 9,9-Bis(4-(2-Hydroxyethoxy) phenyl)Fluorene (BPEF) and 9,9-Bis [3-phenyl-4-(β-Hydroxyethoxy) phenyl]fluorene (BBPEF) were acquired from Xinnuolixing Co., Ltd. (Huanghua, China). The ultra-pure water used in the experiment is deionized water obtained through filtration.

### 2.2. Composite LB Film Preparation

The interface assembly experiment of LB film was carried out by the KSV NIMA LB system at normal temperature and pressure. The LB tank was cleaned with ultrapure water and ethanol. Subsequently, the BPEF and BBPEF were dissolved in chloroform to create the spreading agent solution. Aqueous dye solutions of MB, RhB, and ST at a concentration of 1 × 10^−3^ mol/L were prepared as subphases and then sequentially introduced into the LB tank. By microinjection method, 100 μL 1.0 mg/mL BPEF and BBPEF solution were uniformly added to the subphase surface. A Langmuir monolayer film forms on the subphase surface when the sliding barrier speed is set at 5 mm/min and the surface pressure reaches 20 mN/m. The single-layer film was transferred from the subphase surface to the quartz substrate to finalize the LB film preparation. After the film dried for 30 s, the above steps were repeated to form multilayer LB film on the substrate.

### 2.3. Characterizations

The atomic force microscope (AFM) images were obtained using the Nanoscope model MultiMode 8 Scanning Probe Microscope (VEECO Instruments, Plainview, NY, USA). The morphology of the single-layer film was analyzed by transmission electron microscope (TEM, HT7700, high Technologies Corp, Ibaraki, Japan). The water contact Angle of the film was measured by the Kino SL200KS contact Angle meter (KINO Scientific Instrument Inc., Boston, MA, USA), and the hydrophilicity of the film surface was analyzed. Ultraviolet analysis was performed with an ultraviolet spectrometer (UV-2550, Shimadzu Corporation, Kyoto, Japan). The photoelectrochemical properties of LB composite films were measured by a CHI660 electrochemical workstation (Chenhua Instrument Co., Ltd., Shanghai, China).

## 3. Results and Discussion

**Figure 1:**
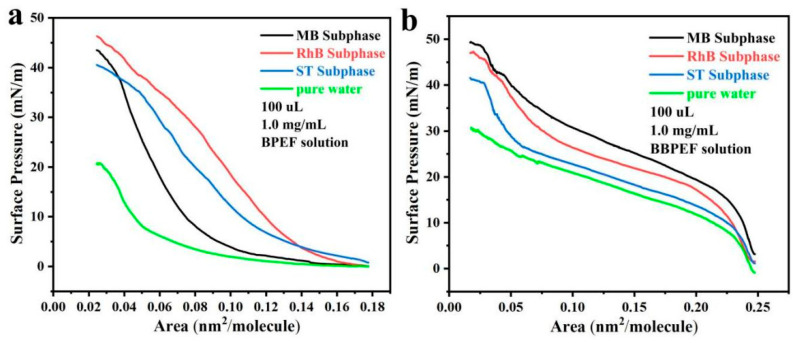
Surface pressure-area isotherms (π-A) of Langmuir films: (**a**) BPEF solution spread on the surface of different subphases; (**b**) BBPEF solution spread on the surface of different subphases.

The BPEF and BBPEF solutions were uniformly diffused over MB, RhB, ST, and pure water subphases in a concentration of 1.0 mg/mL in 100 μL volumes. The resulting surface pressure-area isotherm (π-A) is obtained, as shown in Figure 1. For BPEF molecules, the isotherms started to rise at a surface pressure of 0.08 nm^2^/molecule, 0.14 nm^2^/molecule, and 0.14 nm^2^/molecule in the subphases of MB, RhB, and ST dye solutions, respectively, while the maximum surface pressures reached 45 mN/m, 48 mN/m, and 40 mN/m, respectively. For BBPEF molecules, the isotherms started to rise at a surface pressure of 0.25 nm^2^/molecule, and the maximum surface pressures reached 50 mN/m, 49 mN/m, and 42 mN/m, respectively, in the subphases of MB, RhB, and ST dye solutions. Through data analysis, it can be seen that the two molecules and the three dye subphases can form a good LB monolayer film, and the prepared film can be used for subsequent morphological characterization and performance testing.

**Figure 2:**
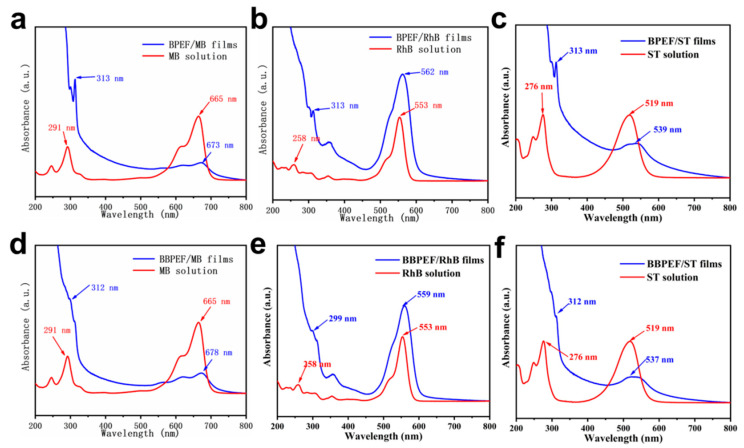
UV–vis spectra of multilayer composite LB films: (**a**) BPEF/MB films; (**b**) BPEF/RhB films; (**c**) BPEF/ST films; (**d**) BBPEF/MB films; (**e**) BBPEF/RhB films; (**f**) BBPEF/ST films.

The UV-VIS absorption spectra of the composite film in Figure 2 demonstrate how the aggregation state of dye molecules greatly affects the spectral characteristics. In Figure 2a, it can be observed that the maximum absorption peak of the composite membrane is redshifted from 665 nm to 673 nm, and the minimum absorption peak is also redshifted, indicating that BPEF interacts with MB molecules to form J-type aggregates. For the BPEF/RhB composite film, it is observed in Figure 2b that the maximum absorption peak is redshifted to 562 nm, and the minimum absorption peak is redshifted to 313 nm relative to the dye, which proves the formation of a J-type aggregate. Figure 2c shows that the maximum absorption peak of the BPEF/ST composite film is red-shifted from 519 nm to 539 nm in the dye solution, further confirming the formation of J-type aggregates. Figure 2d shows that the maximum absorption peak of BBPEF/MB composite film is redshifted to 678 nm, and the minimum absorption peak is blue-shifted to 312 nm. It shows that the interaction between BBPEF and MB molecules promotes the formation of J and H-type aggregates. In Figure 2e, the maximum and minimum absorption peaks of BBPEF/RhB composite films are located at 559 nm and 299 nm, respectively, indicating the existence of J-type aggregates. In Figure 2f, it can be seen that the maximum and minimum absorption peaks of BBPEF/ST composite films have a slight red shift, which once again proves the existence of J-type aggregates.

**Figure 3:**
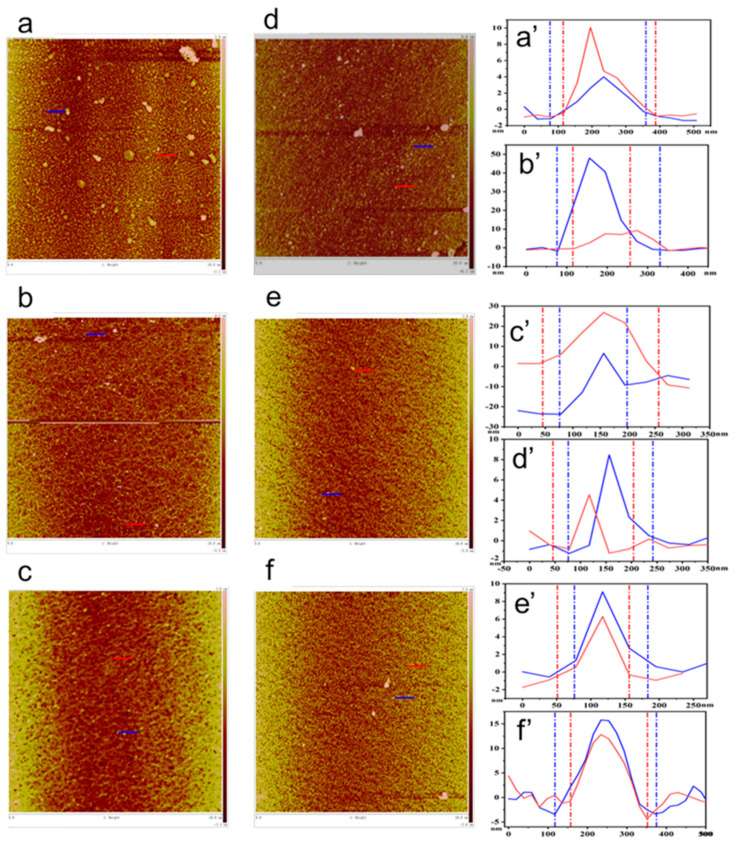
AFM images of monolayer films: (**a**,**a’**) BPEF/MB films; (**b**,**b’**) BPEF/RhB films; (**c**,**c’**) BPEF/ST films; (**d**,**d’**) BBPEF/MB films, (**e**,**e’**) BBPEF/RhB films, (**f**,**f’**) BBPEF/ST films.

Figure 3 shows the AFM structure of the monolayer LB film formed by the interaction of BPEF and BBPEF solutions with three dyes: MB, RhB, and ST. It can show that the composite film is highly uniform and dense, with a thickness of about 20 nm. At a surface pressure of 20 mN/m, Figure 3a,a’ demonstrates that BPEF molecules show a tendency to form dense aggregates in the MB subphase, with a primary height of approximately 10 nm, indicating a robust interaction between BPEF molecules and MB that facilitated the creation of a stable Langmuir film on the subphase surface. This observation of molecular behavior is corroborated by the depiction in Figure 3b,b’,c,c’, where the BPEF/RhB composite film exhibits a primary height of 10 nm, while the BPEF/ST composite film appears to aggregate at the height of 20 nm. The effect of different subphase conditions on the intermolecular interaction was further proved. Figure 3d,d’ depicts the aggregation tendency of BBPEF molecules in the MB subphase, with a predominant height of approximately 8 nm. Figure 3e,e’,f,f’ demonstrate that BBPEF molecules exhibit aggregation tendencies in RhB and ST subphases, resulting in the formation of compact composite films measuring 10 nm and 15 nm in height. Atomic force microscopy images revealed that individual molecules exhibited varying aggregation states on three subsurface layers (MB, RhB, and ST), confirming the successful fabrication of monolayer films.

**Figure 4:**
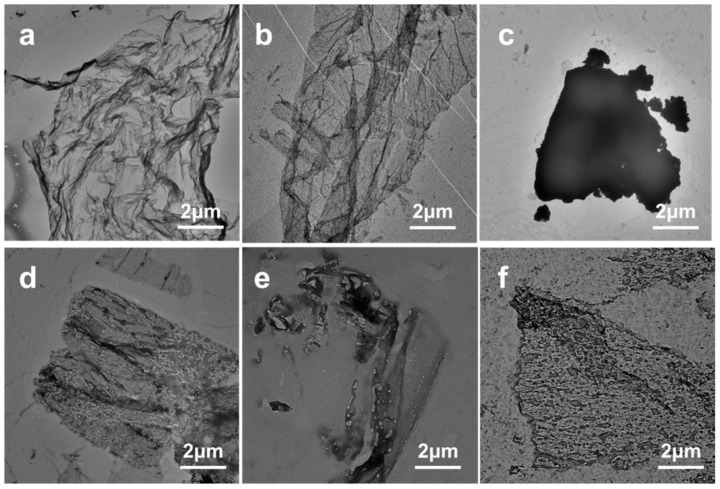
TEM images of the composite films: (**a**) BPEF/MB films; (**b**) BPEF/RhB films; (**c**) BPEF/ST films; (**d**) BBPEF/MB films; (**e**) BBPEF/RhB films; (**f**) BBPEF/ST films.

The TEM images show the morphology of the LB film at the air-liquid interface. Figure 4a shows the layered aggregation and network structure of BPEF molecules at the microscopic scale. The adsorption of MB molecules on the long chain of BPEF resulted in the formation of a relatively dense monolayer with local lamination. In Figure 4b, it is observed that irregular flakes form between BPEF and RhB, and a network of BPEF/MB monolayers exists around them. In addition, the composite film of BPEF with ST shown in Figure 4c exhibits a surface with blocky and reticular structures, distinctly different from the monolayer film morphology of BPEF/MB. Figure 4d illustrates that BBPEF molecules aggregate into sheet-like film structures, demonstrating the formation of BBPEF/MB monolayer LB films. This indicates that MB molecules are adsorbed by BBPEF molecules, forming a relatively dense arrangement of monomolecular layer films. From Figure 4e, it can be concluded that the interaction between BBPEF and RhB results in the formation of irregularly shaped flakes surrounded by chain-like aggregated reticular structures. As shown in Figure 4f, the composite film surface of BBPEF/ST forms blocky thin film reticular structures.

**Figure 5:**
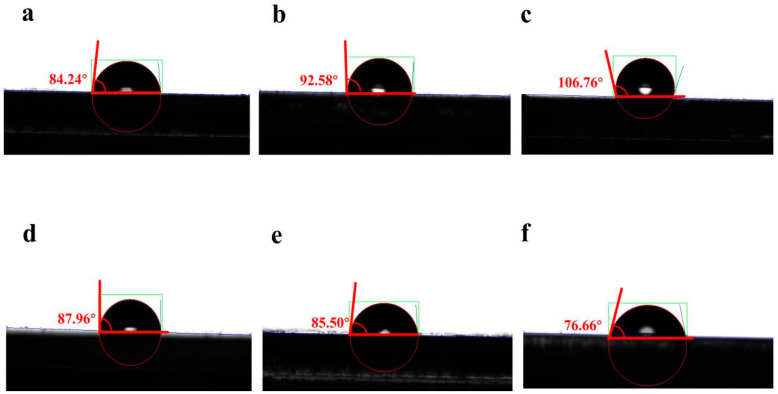
Contact angle test images of the composite films: (**a**) BPEF/MB films; (**b**) BPEF/RhB films; (**c**) BPEF/ST films; (**d**) BBPEF/MB films; (**e**) BBPEF/RhB films; (**f**) BBPEF/ST Films.

The composite film’s surface structure significantly affects the contact angle formed when it comes into contact with water. This study used contact angle measurement techniques to quantitatively analyze the wettability of material surfaces. From Figure 5a–c, it is observed that BPEF molecules exhibit contact angles of 84.24°, 92.58°, and 106.76° in three different subphase environments. In Figure 5d–f, BBPEF molecules display contact angles of 87.96°, 85.50°, and 76.66° under two different subphase conditions. The contact angle test proved that the prepared BPEF/dye films and BPEEF/dye films composite LB films are hydrophobic. The contact angle test showed that the prepared BPEF/dye films and BPEEF/dye films composite LB membranes are hydrophilic.

**Figure 6:**
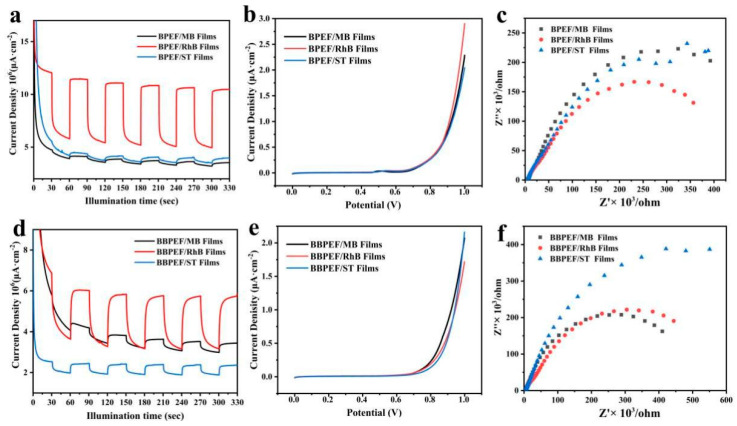
(**a**,**d**) Films I-T curves; (**b**,**e**) films LSV curves; (**c**,**f**) EIS curves of BPEF/dye and BBPEF/dye composite LB films.

Figure 6 illustrates the performance of BPEF/dye and BPEEF/dye composite LB films in the field of optoelectronics. The CHI660 electrochemical workstation was used for the photoelectrochemical test. The electrolyte was a KOH solution with a concentration of 1 M, the Hg/HgO electrode was used as the reference electrode, and the platinum sheet was used as the counter electrode. The light source is a 500 W xenon lamp with a 1.5 g AM filter, and the light intensity I0 value is 100 mW/cm^2^. The EIS measurement is based on an AC voltage amplitude of 10 millivolts. Through repeated testing, it was found that LB films of composite MB, RhB, and ST dyes all exhibit significant photoelectrochemical current responses. Among them, the BPEF/RhB film shows the most prominent photoelectrochemical current intensity. Upon exposure to light, the photoelectrochemical current of all three films quickly reaches peak values, demonstrating the rapid response and stability of BPEF/dye composite LB films as photoanodes to light [22,23]. After light exposure is terminated, the photoelectrochemical current similarly decreases rapidly. The excellent repeatability observed in multiple cycles highlights the feasibility of BPEF/dye films as photoanodes.

Further investigation into the photoelectrochemical conversion efficiency of the composite films was conducted through LSV tests. The BPEF/RhB film shows a higher carrier density, consistent with the results of transient photoelectrochemical current response experiments. Additionally, EIS analysis provides more information about carrier transport efficiency. Data in Figure 6c indicate that the BPEF/RhB film exhibits the lowest impedance compared to other samples, demonstrating its strong carrier migration capability.

## 4. Conclusions

This work successfully prepared LB films of BPEF and BBPEF combined with the dyes ST, RhB, and MB using the LB technique. These films are assembled through weak interactions between BPEF, BBPEF, and dye molecules, showing an adjustable and uniform film structure. This study delved into the morphological characteristics, spectral features, hydrophobicity/hydrophilicity, and photoelectrochemical properties of BPEF/dye and BBPEF/dye composite films. Through the analysis of LSV, I-T, and EIS curves, it is found that the composite films have remarkable photochemical current response and good repeatability. Particularly, BPEF/RhB and BBPEF/RhB composite films exhibited excellent photoelectrochemical response performance. This suggests they have potential applications in solar cells, photodetectors, and other optoelectronic devices. It is expected to be used to develop high-performance sensors for the detection of trace substances or biomolecules in the environment. These findings highlight their broad prospects in photovoltaic applications and provide research directions and methods for the development of multifunctional thin film electronic devices in future research.

## Data Availability

Data presented in this article are available on request from the corresponding author.

## References

[1] Makiura R. (2022). Creation of metal–organic framework nanosheets by the Langmuir–Blodgett technique. Coord. Chem. Rev..

[2] Swierczewski M., Bürgi T. (2023). Langmuir and Langmuir–Blodgett Films of Gold and Silver Nanoparticles. Langmuir.

[3] Qian C., Wang R., Li M., Li X.T., Ge B.C., Bai Z.H., Jiao T.F. (2021). Facile preparation of self-assembled black phosphorus-based composite LB films as new chemical gas sensors. Colloids Surf. Physicochem. Eng. Asp..

[4] Oliveira O.N., Caseli L., Ariga K. (2022). The Past and the Future of Langmuir and Langmuir–Blodgett Films. Chem. Rev..

[5] Caplan M.R., Moore P.N., Zhang S., Kamm R.D., Lauffenburger D.A. (2000). Self-assembly of a beta-sheet protein governed by relief of electrostatic repulsion relative to van der Waals attraction. Biomacromolecules.

[6] Li S.H., Mu J., Wang W.J., Ma S.H., Sun J.L., Chu J.H., Wang W.C. (2004). Polarization of hemicyanine Langmuir-Blodgett films. Chin. Phys. Lett..

[7] Ozbek Z., Erdogan M., Capan R. (2014). Swelling behavior of pyrene-labelled polystyrene LB thin film exposed to various volatile organic vapors. Sens. Actuators B-Chem..

[8] Ozmen M., Ozbek Z., Bayrakci M., Ertul S., Ersoz M., Capan R. (2015). Preparation of Langmuir–Blodgett thin films of calix [6]arenes and p-tert butyl group effect on their gas sensing properties. Appl. Surf. Sci..

[9] Liu X.J., He Y., Zhang G.C., Wang R., Zhou J.X., Zhang L.X., Gu J.M., Jiao T.F. (2020). Preparation and High Photocurrent Generation Enhancement of Self-Assembled Layered Double Hydroxide-Based Composite Dye Films. Langmuir.

[10] Obraztsov I., Noworyta K., Hart A., Gobeze H.B., Kc C.B., Kutner W., D’Souza F. (2014). Langmuir–Blodgett Films of Self-Assembled (Alkylether-Derivatized Zn Phthalocyanine)–(C60 Imidazole Adduct) Dyad with Controlled Intermolecular Distance for Photoelectrochemical Studies. ACS Appl. Mater. Interfaces.

[11] Yamada S., Tasaki T., Akiyama T., Terasaki N., Nitahara S. (2003). Gold nanoparticle-porphyrin self-assembled multistructures for photoelectrochemical conversion. Thin Solid Film..

[12] Stepashkin N.A., Chernenko M.K., Khripun V.D., Ivanov N.S., Sukhodolov N.G. (2018). Electrochemical properties of Langmuir-Blodgett films containing cobalt hexacyanoferrate nanoparticles. Thin Solid Film..

[13] Dähne L., Biller E. (1998). Color Variation in Highly Oriented Dye Layers by Polymorphism of Dye Aggregates. Adv. Mater..

[14] Liu Y.M., Ma K., Jiao T.F., Xing R.R., Shen G.Z., Yan X.H. (2017). Water-Insoluble Photosensitizer Nanocolloids Stabilized by Supramolecular Interfacial Assembly towards Photodynamic Therapy. Sci. Rep..

[15] Alhalili Z., Abdelrahman E.A. (2024). Facile Synthesis and Characterization of Manganese Ferrite Nanoparticles for the Successful Removal of Safranine T Dye from Aqueous Solutions. Inorganics.

[16] Mondal S., Doloi B., Ghosh S. (2013). Spectroscopic studies of interaction of safranine T with ionic surfactants. Fluid Phase Equilibria.

[17] Bar N., Chowdhury P. (2022). A Brief Review on Advances in Rhodamine B Based Chromic Materials and Their Prospects. ACS Appl. Electron. Mater..

[18] Kubra K.T., Salman M.S., Hasan M.N. (2021). Enhanced toxic dye removal from wastewater using biodegradable polymeric natural adsorbent. J. Mol. Liq..

[19] Yao H., Kobayashi S., Kimura K. (2007). Self-assembly of acridine orange dye at a mica/solution interface: Formation of nanostripe supramolecular architectures. J. Colloid Interface Sci..

[20] Kato N., Ikeda S., Hirakawa M., Ito H. (2017). Relationship between degree of polymerization and optical and thermal properties of fluorene in polycarbonate polymers. J. Appl. Polym. Sci..

[21] Peet J., Kim J.Y., Coates N.E., Ma W., Moses L.D., Heeger A.J., Bazan G.C. (2007). Efficiency enhancement in low-bandgap polymer solar cells by processing with alkane dithiols. Nat. Mater..

[22] Ke W.C., Zhang Y., Imbault A.L., Li Y.H. (2021). Metal-organic framework derived iron-nickel sulfide nanorods for oxygen evolution reaction. Int. J. Hydrogen Energy.

[23] Shi G.C., Wang M.L., Zhu Y.Y., Wang Y.H., Ma W.L. (2018). Synthesis of flexible and stable SERS substrate based on Au nanofilms/cicada wing array for rapid detection of pesticide residues. Opt. Commun..

